# Dogs display context-dependent behavioral responses to subtle owner-derived multimodal stimuli

**DOI:** 10.3389/fvets.2026.1916007

**Published:** 2026-08-20

**Authors:** Rio Ikeda, Maaya Saito, Yuki Iwata, Takumi Ozono, Tsige Tadesse Alemayoh, Takatomi Kubo, Ei-Ichi Izawa, Koichi Fujiwara, Toshitaka Yamakawa, Kazunori Ohno, Takefumi Kikusui, Miho Nagasawa

**Affiliations:** 1 Department of Animal Science and Biotechnology, Azabu University, Sagamihara, Japan; 2 Graduate School of Information Sciences, Tohoku University, Sendai, Japan; 3 Tough Cyber-Physical AI Research Center, Tohoku University, Sendai, Japan; 4 Graduate School of Science and Technology, Nara Institute of Science and Technology, Ikoma, Japan; 5 Okinawa Institute of Science and Technology, Onna-son, Japan; 6 Research Institute for Electronic Science, Hokkaido University, Sapporo, Japan; 7 Medilux Research Center, Nara Institute of Science and Technology, Ikoma, Japan; 8 Department of Materials Process Engineering, Nagoya University, Nagoya, Japan; 9 Quadlytics Inc., Kyoto, Japan

**Keywords:** dog, emotional cue, heart rate variability, human–dog interaction, physiological synchrony, stress context

## Abstract

**Introduction:**

Dogs (*Canis familiaris*) can discriminate explicit human emotions using various sensory modalities. However, their sensitivity to subtle human-derived emotional cues accompanied by physiological changes, but lacking clear behavioral cues, remains poorly understood. This study investigated whether dogs could perceive owner-derived emotional cues when suppressing explicit expressions.

**Methods:**

Owners were assigned to either stress or relaxation conditions designed to induce stress or relaxation, respectively. Following the experimental manipulation, facial video, voice, and olfactory samples were collected during a standardized reading task and simultaneously presented to the dogs. The behavioral responses and heart rate variability (HRV) of the dogs during stimulus presentation were recorded.

**Results:**

Owners exhibited elevated physiological arousal during both the control and stress induction periods, whereas negative mood increased only during stress induction. In response to these owner-derived stimuli, dogs under the stress condition displayed greater stimulus-directed attention toward control-derived stimuli than toward stress-derived stimuli. No clear effects of condition or stimulus type on dog HRV were observed; however, SDNN-based physiological synchrony between owners and dogs tended to increase with stress-derived stimuli.

**Discussion:**

Our results suggest that dogs respond to complex owner-derived stimuli in a context-dependent manner. These findings suggest that dogs may respond to subtle combinations of owner-derived cues that reflect physiological arousal, subjective emotional state, and task-related influences.

## 1 Introduction

Dogs (*Canis familiaris*), the earliest domesticated animals, are thought to have diverged approximately 33,000–11,000 years ago (1, 2). This domestication process resulted in significant morphological and neurophysiological changes. Notably, a dog’s brain weight relative to its body weight is approximately three-quarters that of a wolf (3), and some sensory capacities, such as olfaction and audition, have been reported to decline compared to their wild ancestors (4). However, these changes are deeply linked to paedomorphosis (neoteny) associated with domestication, involving delayed developmental processes and the retention of strong social receptivity (5). Although dogs may have lost some of the acute sensory skills required in wild environments, they are believed to have evolved to allocate more cognitive resources toward maintaining emotional and social relationships with humans.

Dogs have developed specialized facial muscle anatomy, such as the levator anguli oculi medialis, to produce expressions that elicit caregiving responses in humans, an evolutionary adaptation specifically for interspecific nonverbal communication (6). Selection for reduced fear and aggression toward humans (7) likely facilitated the development of human-like social cognitive abilities as a byproduct of selection for emotional reactivity (8). A dog’s gaze functions as an attachment behavior that modulates the human endocrine system, leading to the establishment of interspecific bonding (9, 10). Dogs demonstrate an exceptional ability to understand human social cues, such as gaze and pointing, and adapt their behavior accordingly (11, 12). A recent study has shown that these social cognitive abilities are associated with genetic polymorphisms related to oxytocin receptors and cortisol secretion (13). Consequently, interspecific social cognition is considered a key component of evolutionary adaptation in long-standing cooperative relationships with humans.

Recent ethological and neuroscientific studies have shown that dogs can discriminate between explicit human emotions, such as joy, anger, and fear, using various sensory modalities. They adjust their looking behavior and social responses based on human emotional valence (14–17) and can identify emotional states through vocal prosody (18). Furthermore, emotional contagion has been reported, in which the perception of human emotions extends to a dog’s physiological state. Synchrony in heart rate variability (HRV) has been confirmed under negative circumstances (19). Neuroimaging studies using fMRI further support this, reporting that reward-related regions such as the caudate nucleus are activated when dogs hear positive words spoken with positive intonation, indicating a neural basis for processing socioemotional cues as having value (20).

In addition to the perception of explicit emotional expressions, dogs possess remarkable sensitivity to subtle physiological changes that are often imperceptible to humans. Chemical cues in human body odor affect the behavioral and physiological responses of dogs, suggesting that dogs process emotional information by integrating multiple sensory modalities (21–23). Furthermore, dogs can detect the specific scent of an impending epileptic seizure several minutes before its onset (24). They can also distinguish volatile organic compounds in human breath or urine to identify early signs of various cancers (25, 26). These findings indicate that dogs can react to biological changes that humans may not perceive themselves.

However, most previous studies have focused on explicit emotional expressions in which facial or vocal changes are clearly manifested. In contrast, in daily life, humans often suppress or regulate their emotional expression, resulting in minimal overt changes despite significant internal physiological arousal. The ability of dogs to perceive these subtle or implicit emotional states—such as mild stress or tension that lack clear behavioral cues but are accompanied by physiological changes—remains poorly understood. Perceiving such subtle shifts is crucial for understanding the depth of the human-dog bond, as it requires dogs to resolve the potential incongruence between a human’s internal state and outward appearance.

Therefore, the present study aimed to investigate whether dogs can perceive subtle emotional changes in their owners when explicit cues are minimized using an adapted paradigm based on Katayama et al. (19). We established two independent experimental conditions: a stress condition induced by the Trier Social Stress Test (TSST) and a relaxation condition induced by viewing relaxing videos. Following these inductions, we collected multimodal stimuli (video of the face, audio, and sweat/saliva as olfactory cues), while the owners performed a neutral task (reading a fixed sentence aloud) to simulate the suppression of overt emotional expression. These stimuli were presented simultaneously to the dogs. By evaluating dogs’ behavioral responses and HRV, we aimed to clarify how dogs respond to subtle emotional fluctuations that are not present as clear behavioral stress responses. This study aims to provide new insights into social cognition and physiological coordination in the human-dog relationship, particularly regarding dog responses to subtle owner-derived multimodal cues associated with different emotional contexts through multi-sensory integration.

## 2 Materials and methods

### 2.1 Subjects

In total, 35 dog–owner pairs participated in this study (Supplementary Table S1). The participants were recruited at events that targeted dogs and their owners. Prior to participation, owners were verbally asked to confirm that both owners and their dogs were in good health with no chronic illnesses, injuries, or other medical conditions; this confirmation was set as the inclusion criterion. All experiments were conducted in experimental rooms at Azabu University between March 20, 2024, and March 27, 2025. The owners were informed in advance that their dogs could participate only if they did not exhibit fear or aggression toward unfamiliar places or people. The owners were also informed that the experiment would be immediately terminated if their dog showed signs of excessive stress, including persistent trembling, drooling, barking, growling, refusal to leave the owner’s side, and avoidance of the experimenters or experimental apparatus, or if the owner judged that their dog was stressed and requested termination. In addition, eight healthy adults (mean age ± SD: 20.0 ± 1.13 years) participated as third-party evaluators. None of the third parties had any prior acquaintance with the owners.

All experimental procedures were approved by the Animal Ethics Committee of Azabu University (#230318–21) and the Ethics Committee for Medical and Health Research Involving Human Subjects of Azabu University (#052). Informed consent was obtained from the owners, who could stop participating at any time for any reason. Each human participant was compensated with a 2,000-yen Amazon gift card, and the participating dogs received dog treats.

### 2.2 Apparatus

**Figure 1:**
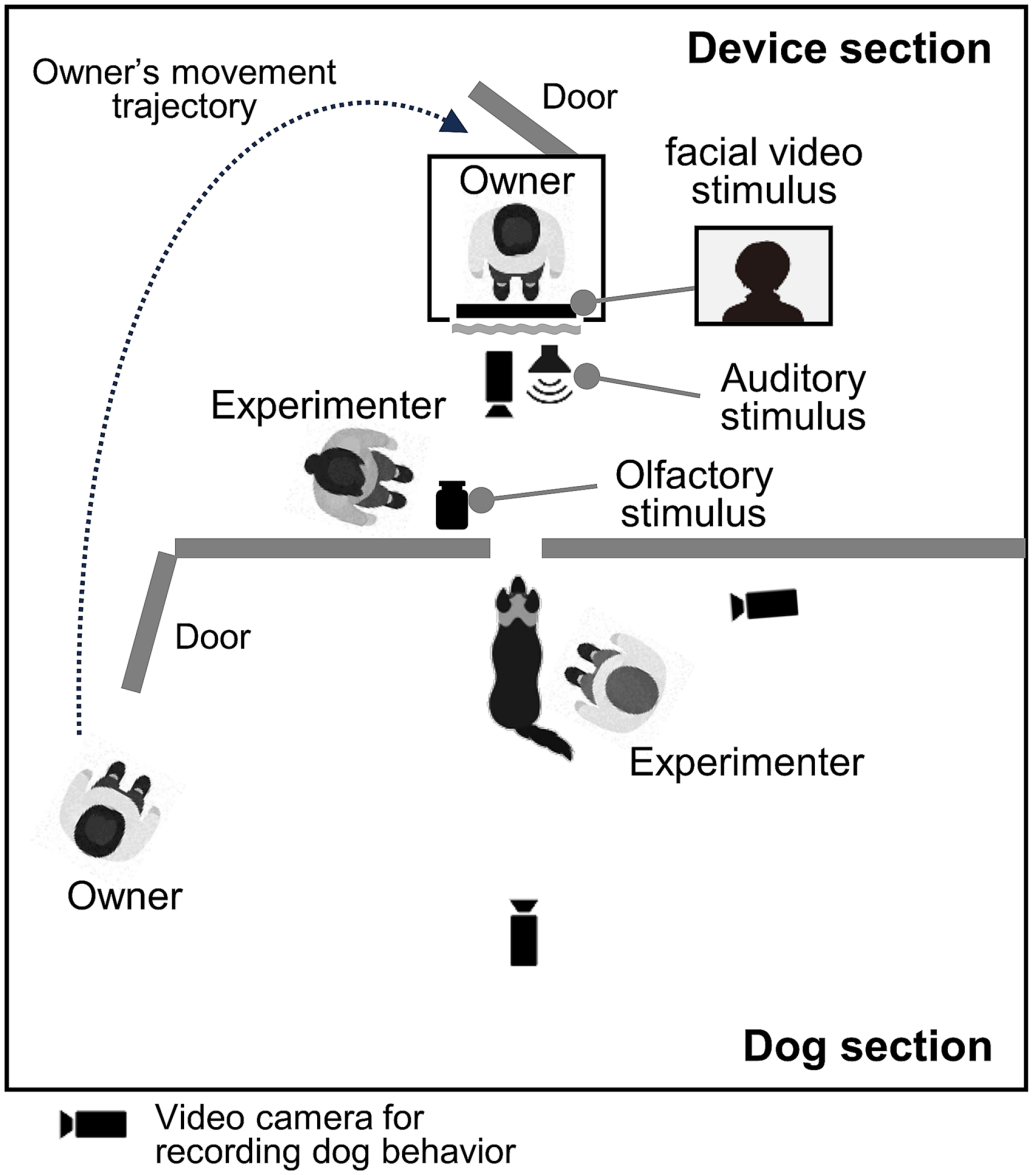
The experimental setup during the dog phase. The dogs observed a stimulus presentation device from an adjacent section through a gap in the partition. The device presented multimodal stimuli (visual, auditory, and olfactory) derived from the owner phase, while the owner remained inside the apparatus but out of the dog’s direct view.

The experimental setup is shown in Figure 1. The experiment consisted of two phases conducted in separate rooms: an owner phase for emotional induction and stimulus acquisition and a dog phase for stimulus presentation.

The owner phase was conducted in a room measuring 300 cm × 160 cm. The owner sat on a chair facing the two experimenters. To standardize the visual background, a partition covered with white cloth was placed behind the owner during the close-up video recording. One camera (HDR-AS50; Sony Corp., Tokyo) was positioned behind the experimenter to record the entire room, while another camera (Handycam FDR-AX700; Sony Corp., Tokyo) was placed approximately 1 m in front of the owner to record chest-up footage.

In the dog phase, a single room measuring 1,328 × 1,060 cm was divided into two adjacent sections using a partition. One section contained the stimulus presentation device (device section), whereas the other served as the dog section. A gap (20–30 cm wide) was left at the center of the partition, allowing the dog to access the device visually from the dog section. The stimulus presentation device was a box-shaped booth large enough for a person to enter and sit inside and was constructed using a commercially available cardboard soundproof booth (Danbocchi Wide type, Kanda Sangyo Co., Ltd., Fukushima). The booth was equipped with a 31.5-inch 4 K monitor (32UP550, LG Electronics Japan, Tokyo) and a monaural speaker (RL906, Musikelectronic Geithain, Germany), which were used to present the owner’s prerecorded facial videos and voices obtained during the owner phase. A pair of curtains was installed over the window where the monitor was placed and was opened remotely during the stimulus presentation trials. The monitor could switch between a prerecorded video and a live video feed from inside the booth, so that the dog could recognize that the owner was inside the device. The device was positioned 1.5 m from the gap. The dog’s behavior was recorded using three cameras positioned in front of the dog, on the dog’s right side, and behind the dog (HDR-AS50 and Handycam FDR-AX700; Sony Corp., Tokyo).

The cardiac activity of the owners and dogs was recorded throughout the experiment using a wearable heart rate monitor (FAROS360; Bittium Corp., Oulu, Finland). Disposable ECG electrodes (M-VITROD; Nihon Kohden Corp., Tokyo, Japan) were attached using a standard three-lead configuration before starting the experiment.

### 2.3 Procedure

**Figure 2:**
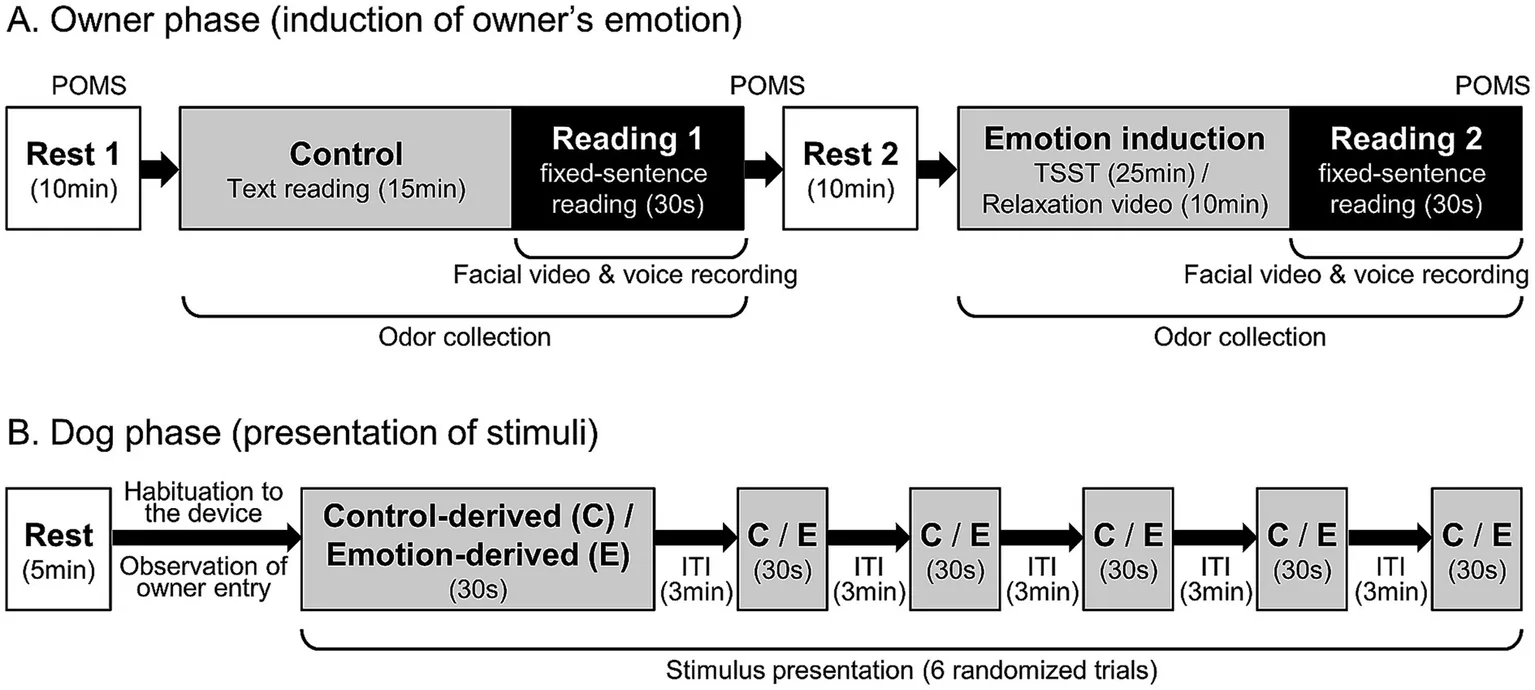
Experimental procedure. **(A)** Owner phase: Owners completed the same procedure under two conditions (stress or relaxation), consisting of a control period and an emotion induction period, each followed by a fixed-sentence reading (Reading 1 and Reading 2) which were used for stimulus acquisition. **(B)** Dog phase: After a rest and habituation period, dogs were presented with multimodal stimuli (visual, auditory, and olfactory) derived from the owner’s Reading 1 (control-derived) and Reading 2 (emotion-derived) periods across six trials.

The experiment consisted of two phases: (1) the owner phase, in which emotional states were experimentally induced and visual, auditory, and olfactory stimuli were collected from the owners; and (2) the dog phase, in which the collected stimuli were presented to the dogs (Figure 2).

#### 2.3.1 Owner phase: owner emotional induction

##### 2.3.1.1 Physiological setup and pre-experimental controls

The owners were instructed to refrain from consuming potentially stimulating substances (e.g., strong-smelling foods or beverages, alcohol) and from using strongly scented products (e.g., fabric softener, perfume, tobacco) the day before the experiment. They were also asked to avoid excessive contact with the animals, which could result in odor transfer. For experiments starting at 9:00 a.m., owners were instructed to abstain from food and beverages other than water after 9:00 p.m. the previous evening; for experiments starting after noon, intake was restricted beginning 3 h prior to the experiment. Immediately before the experiment, the owners brushed their teeth without toothpaste. To minimize the influence of clothing-related odors, the owners changed into new T-shirts provided by the experimenters. A heart rate monitor was attached to the owners using the three-lead method, following Katayama et al. (19).

##### 2.3.1.2 Emotional induction

Each owner completed either the stress or relaxation condition in a between-subjects design. Owners were randomly assigned to one of the two conditions. Both conditions followed the same overall temporal structure and consisted of two experimental periods (a control period and an emotional induction period), each preceded by a resting period and followed by a fixed-sentence reading period.

The owners first completed a 10-min resting period (Rest 1) to establish a baseline. This was followed by a control period (Control), consisting of 5 min of silent reading and 10 min of reading simple material aloud. Immediately after the Control period, the owners read aloud a fixed sentence (consisting of the 50 Japanese hiragana syllables and the 26 letters of the English alphabet) for 30 s (Reading 1). Subsequently, the owners completed a second 10-min rest period (Rest 2). They then underwent an emotion induction period (Emotion), followed by another 30-s reading of the same fixed sentence (Reading 2). The reading periods (Reading 1 and Reading 2) served as standardized periods for stimulus acquisition and physiological analysis. Owners were instructed to read the sentences naturally while avoiding deliberate changes in facial expression, voice, or prosody. Following data collection, the experimenter reviewed the recordings and confirmed that no evident condition-related differences were observed in the overt facial expressions or vocal delivery. The video and audio recordings obtained during these periods were used as visual and auditory stimuli in subsequent dog and third-party experiments. The emotion induction procedure differed by condition; in the stress condition, a modified version of the Trier Social Stress Test (TSST) was administered (19). The owners first read and memorized a technical text for 5 min. They were then asked to explain the content of the text to the two experimenters for 10 min, while referring to the text as little as possible. Finally, they performed a mental arithmetic task for 10 min. In the relaxation condition, the owners watched a relaxation video for 10 min during the emotion induction period.

Underarm sweat and saliva samples were collected from the owners and used as olfactory stimuli in the dog phase. Sweat samples were collected by placing cotton pads (4 × 4 cm) under the owner’s armpits prior to each resting period (i.e., before Rest 1 and Rest 2). The armpit was wiped with a cleansing pad before cotton was placed. The cotton pads were replaced with new ones after the completion of Reading 1. Saliva samples were collected after completion of Reading 1 and Reading 2 by allowing the saliva to flow through a saliva collection aid (SCA; Salimetrics LLC, Carlsbad, California, USA) into sterile 2-mL tubes. Saliva was collected using either Salimetrics Cryovial 2-mL tubes (5004.01) or sterile 2-mL microtubes (1–1,600-04, AS ONE Corp., Osaka), with a total volume of approximately 2 mL obtained per sampling. Because the dog phase began immediately after stimulus acquisition, sweat and saliva samples intended for presentation to dogs were kept at room temperature and presented without freezing or long-term storage. In addition, portions of both sweat and saliva samples were stored at −20 °C for subsequent component analysis (results not shown).

Throughout this paper, stimuli obtained during the Control period (Reading 1) are referred to as control-derived stimuli, whereas those obtained after the emotion-induction period (Reading 2) are referred to as emotional-induction-derived stimuli. In the Results and Discussion section, these are referred to as stress-derived or relaxation-derived stimuli based on the experimental conditions.

To assess the effectiveness of emotional induction, owners completed the Profile of Mood States, 2nd Edition (POMS2) at the beginning of the experiment and after completing Reading 1 and Reading 2.

#### 2.3.2 Dog phase: dog stimulus presentation

##### 2.3.2.1 Physiological recording and habituation

The dogs were fitted with a heart rate monitor identical to that used for human participants. Following a previous study (19), the M–X lead configuration was adopted to attach the electrodes to the dogs. After attachment of the heart rate monitor, the dogs underwent a 15-min habituation period to acclimate to both the equipment and experimental environment. During this period, the dogs were allowed to freely explore the laboratory with their owners. To habituate to the stimulus presentation device, a stimulus consisting of a chest-up video image and audio recording of a person other than the owner was presented twice prior to the start of the experimental trials.

##### 2.3.2.2 Experimental procedures

After a 5-min period of quiet rest, during which the dog and owner stayed together in the dog section, the owner left the dog in this section and moved to the device section through a door in the partition. One experimenter remained in the dog section and served as a handler to assist with restraint if necessary, whereas the second experimenter operated the stimulus presentation device in the device section. The stimulus presentation device alone was then presented to the dog for 30 s to allow habituation to the device. During this period, the owner remained hidden behind a small partition placed beside the device to ensure that the dog could not see the owner.

Before each trial, the owner was briefly shown entering the device through the door on the back of the device for 30 s to attract the dog’s attention. After entering, the owner remained seated quietly inside the device. Subsequently, visual and auditory stimuli were simultaneously presented to the dog for 30 s from the device. Olfactory stimuli, consisting of combined underarm sweat and saliva samples, were placed in a wide-mouth jar immediately behind the gap on the device-section side of the partition, where they were not visible to the dog. The lid of the jar was opened by the device operator for 30 s simultaneously with the visual and auditory stimuli.

Each stimulus set was derived from the fixed-sentence reading periods conducted during the owner phase, followed by the control period (Reading 1) and the emotional induction period (Reading 2). For each dog, stimulus sets from Reading 1 and Reading 2, corresponding to the owner’s assigned condition, were each presented three times, resulting in six trials. The order of stimulus presentation was randomized across the trials. A 3-min inter-trial interval was provided between trials, during which the owner returned to the dog section and was allowed to interact freely with the dog. At the beginning of the next trial, the owner moved to the device section, entered the apparatus, and presented the next stimulus. This sequence was repeated until all the trials were completed. To maintain double-blind conditions, the experimenters were not informed whether the stimuli presented in each trial were from Reading 1 or Reading 2.

After completion of six trials, the device alone was presented again, accompanied by a brief demonstration of the owner entering the apparatus, and the experiment was concluded.

#### 2.3.3 Stimulus presentation experiment with human participants

To assess whether unfamiliar human observers could infer the owners’ emotional states from the owner-derived stimuli, a stimulus presentation experiment was conducted with healthy third-party participants. This experiment was conducted immediately after the completion of the corresponding dog stimulus presentation phase with the same set of owner-derived stimuli that had been presented to the dogs. These evaluations were used to assess whether the emotional properties of the stimuli were perceptible to unfamiliar human observers.

Third-party participants wore heart rate monitors identical to those used by owners and dogs. The participants were seated in the dog section of the experimental room and viewed the stimulus presentation device from the same location and perspective as the dogs. Using the same stimulus presentation apparatus and largely the same procedure as in the dog experiment, the three types of stimuli (visual, auditory, and olfactory) obtained during the owner phase were presented simultaneously for 30 s per trial. Unlike the dog experiment, the demonstration of the owner entering the device prior to stimulus presentation was omitted.

Immediately after each trial, participants evaluated the emotional valence and arousal of the presented stimuli using a Self-Assessment Manikin (SAM) (27).

### 2.4 Analysis

#### 2.4.1 Heart rate variability

The ECG data recorded using the FAROS360 device were processed using a proprietary MATLAB script to detect R-waves and calculate the RR intervals. R-wave detection errors were visually inspected using electrocardiogram waveforms. Segments in which the R-wave detection was unreliable because of noise or other artifacts were treated as missing values. Data were excluded from further analysis if over 10% of values were missing in each analysis window, as described below. The final sample sizes for each analysis are summarized in Supplementary Table S3.

The extracted RR interval data were subjected to time-domain heart rate variability (HRV) analysis. For owners, the analysis window was set to 120 s with a 2-s shift for longer periods (Rest, Control, and Emotion). However, for the 30-s Reading periods, a shorter analysis window of 15 s with a 2-s shift was applied. Similarly, for dogs and third-party participants, a shorter analysis window of 15 s with a 2-s shift was used to enable HRV calculation during stimulus presentation. The following HRV parameters were calculated: mean RR interval (meanNN); standard deviation of RR intervals (SDNN), which reflects overall autonomic nervous system activity; and root mean square of successive differences (rMSSD), which is an index of parasympathetic activity. For owners, the average HRV values were calculated separately for six periods: Rest1, Control, Reading 1, Rest 2, Emotion, and Reading 2. For dogs and third-party participants, the average HRV values were calculated for the resting period before the start of the experiment and for each of the six stimulus presentation trials. These values were used for the subsequent statistical analyses. For third-party participants, however, HRV data were not subjected to statistical analysis because a large amount of missing data prevented reliable estimation of the HRV indices. Consequently, third-party HRV results were not reported.

#### 2.4.2 Dogs’ behavior

Behavioral variables were defined *a priori* to represent general, stress-related, and stimuli-directed exploratory behaviors. As body shaking, yawning, and licking were observed only a few times or not at all, these behaviors were excluded from the statistical analyses. All of the remaining predefined behavioral variables were included in the statistical analyses, and the complete results are provided in the Supplementary materials.

The dogs’ behavior during stimulus presentation was recorded simultaneously from three angles (front, side, and rear). The recordings were integrated into a single video for behavioral analysis, allowing the behaviors to be comprehensively evaluated across viewpoints. Behavioral coding was conducted using the BORIS software (version 8.22.17) (28), and each behavioral item was quantified in terms of duration or frequency. Each behavioral item was quantified separately for each 30-s stimulus presentation trial. The definitions of each behavior are listed in Supplementary Table S2. Behavioral coding was performed independently by two trained coders. Intercoder reliability was assessed using data from a subset of six dogs (approximately 20% of the sample). For occurrence-based behaviors, Cohen’s kappa coefficients were calculated based on the presence or absence of behaviors across observation segments and indicated perfect agreement between coders (Cohen’s *κ* = 1.00). For duration-based behaviors, total durations across six 30-s segments (180 s per dog) were compared using Bland–Altman analyses, revealing mean differences between coders of no more than 1% of the total observation time.

#### 2.4.3 Questionnaire-based assessments

To assess the effectiveness of emotional induction in the owner phase, owners completed the POMS2 at the start of the experiment and after each reading period. The Total Mood Disturbance (TMD) T-score was used as an index of negative mood. The SAM was used to allow third-party participants to evaluate the emotional valence and arousal of the stimuli presented to dogs. Ratings were made on a 9-point scale after each 30-s stimulus presentation.

#### 2.4.4 Statistical analysis

##### 2.4.4.1 Owner phase analysis

Physiological and subjective data obtained from the owners were analyzed using generalized linear mixed models (GLMMs) with a gamma error distribution and a log link function. HRV data obtained during the owner phase were analyzed using GLMMs. The dependent variables were the mean HRV values calculated during the predefined assessment periods. The fixed effects included Condition (stress vs. relaxation; between-subjects), Period (four levels: Rest 1, Control, Rest 2, and Emotion; within-subjects), and their interaction (Condition × Period). The POMS scores obtained from the owners were analyzed using the same modeling framework. The dependent variable was the TMD T-score assessed at predefined periods. Fixed effects included Condition (stress vs. relaxation), Period (three levels: Rest 1, after Reading 1, and after Reading 2), and their interactions. In both analyses, owner ID was included as a random effect to account for repeated measurements within individuals.

##### 2.4.4.2 Dog phase analyses

For the dog phase, HRV and behavioral measures were averaged separately for each stimulus presentation, and each presentation was treated as one trial for statistical analyses. Dog HRV and behavioral data were analyzed using GLMMs with a gamma error distribution and a log-link function. The dependent variables were physiological measures (heart rate and mean HRV values), and behavioral measures were calculated for each trial. The fixed effects included the Condition (stress vs. relaxation; between-subjects), Stimulus-type (control-derived vs. emotional-induction-derived stimulus; within-subjects), and their interactions. The Dog ID was included as a random effect.

Third-party evaluations of owner-derived stimuli assessed using the SAM were analyzed using the same statistical framework. The dependent variable was the SAM rating assigned to the stimuli presented to the dogs. Fixed effects included Condition and Stimulus-type, as well as their interaction, and rater ID was included as a random effect.

##### 2.4.4.3 Correlation analysis between owner and dog HRV

To examine whether the coupling between the autonomic responses of owners and dogs differed across experimental conditions, correlation analyses were performed using HRV time-series data. Correlation analyses were not performed for HR, as HR is mathematically the inverse of the mean NN interval; therefore, HR-based correlations would be identical to those obtained for the mean NN. For owners, the HRV values obtained during each fixed-sentence reading period (Reading 1 and Reading 2) were used. HRV during the reading period was analyzed as time-series data derived using sliding analysis windows, yielding approximately 14–15 data points per reading period. For dogs, the HRV values obtained during the first presentation of each stimulus type (control- or emotional-induction-derived) were used. Dog HRV was analyzed as time-series data, yielding approximately 14–15 data points per trial. For each owner–dog pair, correlation coefficients were calculated between the owner and dog HRV time series.

These correlation coefficients were analyzed using linear mixed-effects models (LMMs), assuming a normal error distribution with an identity link function. For correlation analyses, the factor representing stimulus origin was defined as Stimulus-origin, referring to the owner’s physiological state during stimulus acquisition (Reading 1 vs. Reading 2) and the corresponding stimulus type presented to the dogs (control-derived vs. emotional-induction-derived stimulus). Condition (stress vs. relaxation), Stimulus-origin, and their interaction were included as fixed effects. Individual ID was included as a random effect to account for repeated measurements within pairs.

##### 2.4.4.4 General statistical procedures

For all GLMM and LMM analyses, the best model was selected by excluding explanatory variables from the full model based on the lowest Akaike’s information criterion (AIC) values. The significance of the fixed-effect independent variables was determined using an F-test. Model assumptions were evaluated by visually inspecting quantile–quantile plots and residual vs. fitted plots of all final models. All models were converged, and no clear deviations from the distributional assumptions were detected. To evaluate the model fit, marginal and conditional R^2^ values were calculated for the final mixed models. Marginal R^2^ represents the proportion of variance explained by fixed effects, whereas conditional R^2^ represents the proportion of variance explained by both fixed and random effects (29). The GLMM analyses were conducted using IBM SPSS Statistics (version 27). To examine the significant differences among the variables of a fixed effect, *post hoc* pairwise contrasts of the estimated marginal means were conducted using Tukey’s correction. Marginal and conditional R^2^ values were calculated using R 4.5.2 (30) and the ‘MuMIn’ package (31).

## 3 Results

### 3.1 Owner’s physiological and subjective data

A GLMM on the owners’ heart rate (HR) selected the full model as the best model, with a significant main effect of Period (*F* = 9.810, df1 = 3, df2 = 95, *p* < 0.001) as well as a significant Condition × Period interaction (*F* = 9.437, df1 = 3, df2 = 95, *p* < 0.001). To compare the periods under each condition, stratified GLMMs were conducted separately for the datasets of the stress and relaxation conditions. In both conditions, a significant effect of the Period was found (stress: *F* = 7.537, df1 = 3, df2 = 37, *p* < 0.001; relaxation: *F* = 12.265, df1 = 3, df2 = 58, *p* < 0.001). In the stress condition, no significant difference in HR between the Control and Emotion periods (*t* = 0.233, *p* = 0.817). However, HR in both the Control and Emotion periods was significantly higher than that in Rest 1 (vs. Control, *t* = 3.725, *p* < 0.001; vs. Emotion, *t* = 3.627, *p* < 0.001) and Rest 2 (vs. Control, *t* = 2.957, *p* = 0.005; vs. Emotion, *t* = 2.772, *p* = 0.009). The HR at Rest 1 did not differ significantly from that at Rest 2 (t = 0.623, *p* = 0.537). In the relaxation condition, pairwise comparisons revealed that the HR in Control period was significantly higher than that in Rest 1 (*t* = 2.041, *p* = 0.046), Rest 2 (*t* = 4.174, *p* < 0.001), and the Emotion period (*t* = 5.575, *p* < 0.001). The HR in Rest 1 was significantly higher than that in Rest 2 (*t* = 2.158, *p* = 0.035) and the Emotion period (*t* = 3.663, *p* = 0.001). No significant difference was observed between Rest 2 and the Emotion period (*t* = 1.606, *p* = 0.114).

A similar pattern was observed for meanNN. A GLMM on meanNN selected the full model with significant effects of Period (*F* = 9.917, df1 = 3, df2 = 95, *p* < 0.001) and Condition × Period interaction (*F* = 9.499, df1 = 3, df2 = 95, p < 0.001). Follow-up stratified GLMM analyses revealed significant effect of period in both conditions (stress: *F* = 7.674, df1 = 3, df2 = 37, *p* < 0.001; relaxation: *F* = 12.274, df1 = 3, df2 = 58, p < 0.001). Pairwise comparisons in the stress condition exhibited no differences between the Control and Emotion periods (t = 0.322, *p* = 0.749), or between Rest 1 and Rest 2 (*t* = 0.668, *p* = 0.508), whereas mean NNs of Control and Emotion periods were shorter than those of Rest 1 and 2 (Control vs. Rest 1, *t* = −3.830, *p* < 0.001; Control vs. Rest 2, *t* = −2.988, *p* = 0.005; Emotion vs. Rest 1, *t* = −3.628, *p* = 0.001; Emotion vs. Rest 2, *t* = −2.712, *p* = 0.010). These results in the stress condition suggest that meanNN decreased during the Control and Emotion periods compared with the preceding rest period. In the relaxation condition, meanNNs in Rest 1 and Control period were shorter than Rest 2 and Emotion period (Rest 1 vs. Rest 2, *t* = −2.206, *p* = 0.031; Control vs. Emotion, *t* = −5.537, *p* < 0.001; Rest 1 vs. Emotion, *t* = −3.718, *p* < 0.001; Control vs. Rest 2, *t* = −4.142, *p* < 0.001), while no differences were found between corresponding Rest and intervention periods (Control vs. Rest 1, *t* = 1.959, *p* = 0.055; Emotion vs. Rest 2, *t* = 1.626, *p* = 0.109). These results in the relaxation condition suggest that meanNN increased from the Rest 1 and Control periods to the Rest 2 and Emotion periods but did not change in response to the intervention periods.

**Figure 3:**
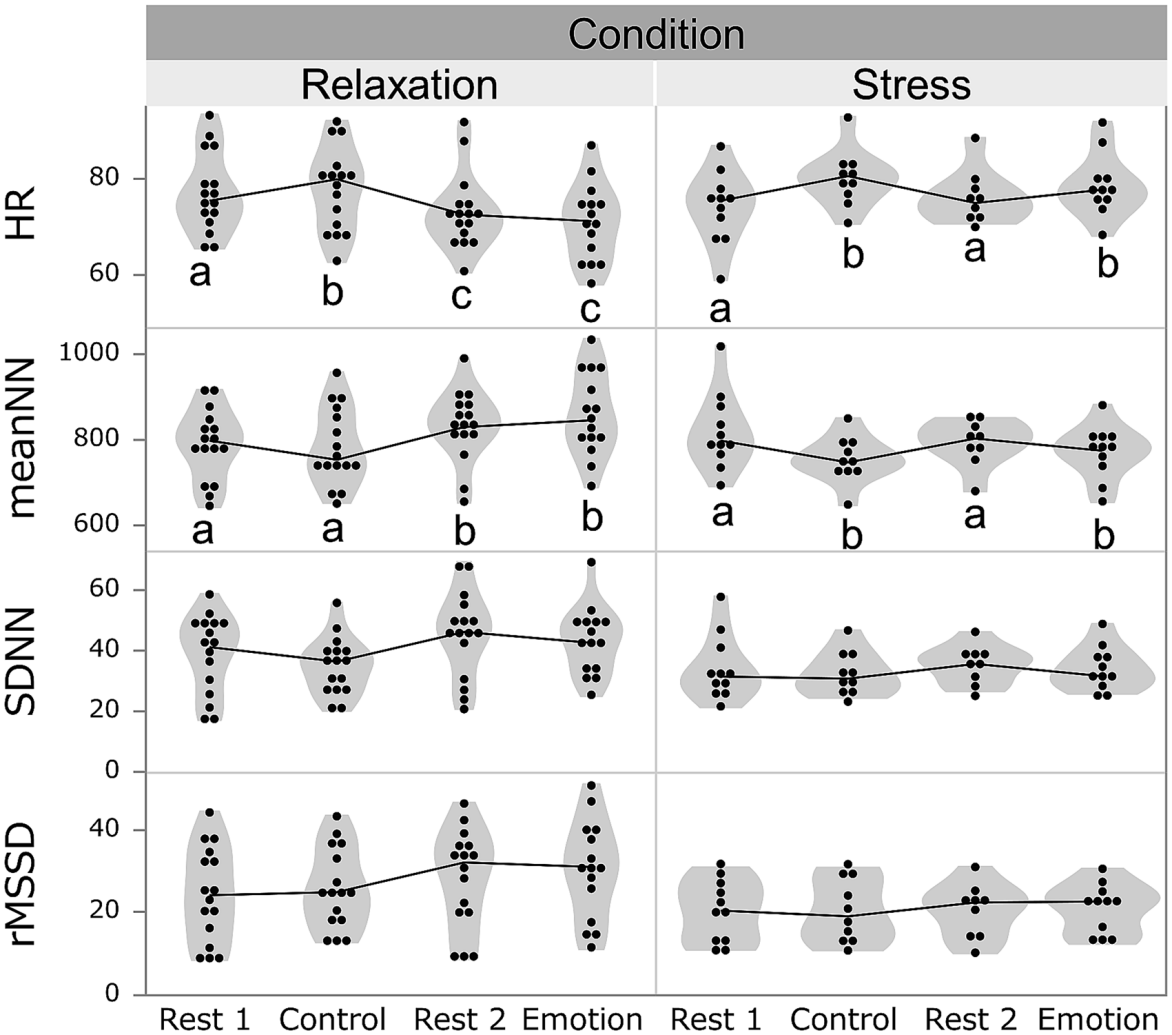
Owner’s HRV. Violin plots show the distribution of HRV indices. Within each panel, the groups that did not share a common letter differed significantly from one another.

For the SDNN, the best model included only the Period variable with a significant effect (*F* = 7.861, df1 = 3, df2 = 99, *p* < 0.001). Pairwise comparisons showed that SDNN was lower in Rest 1 than Rest 2 (*t* = −3.087, *p* = 0.003) and also lower in the Control period than the Emotion period (*t* = −2.986, *p* = 0.004) with no differences between corresponding Rest and intervention periods (Rest 1 vs. Reading 1, *t* = 1.468, *p* = 0.145; Rest 2 vs. Reading 2, *t* = 1.490, *p* = 0.139). These suggest an increase in SDNN in the later periods. No significant effects were found for the rMSSD. All owner HRV data are shown in Figure 3.

The results of the GLMM analyses of the HRV indices are presented in Supplementary Tables S3–S6.

**Figure 4:**
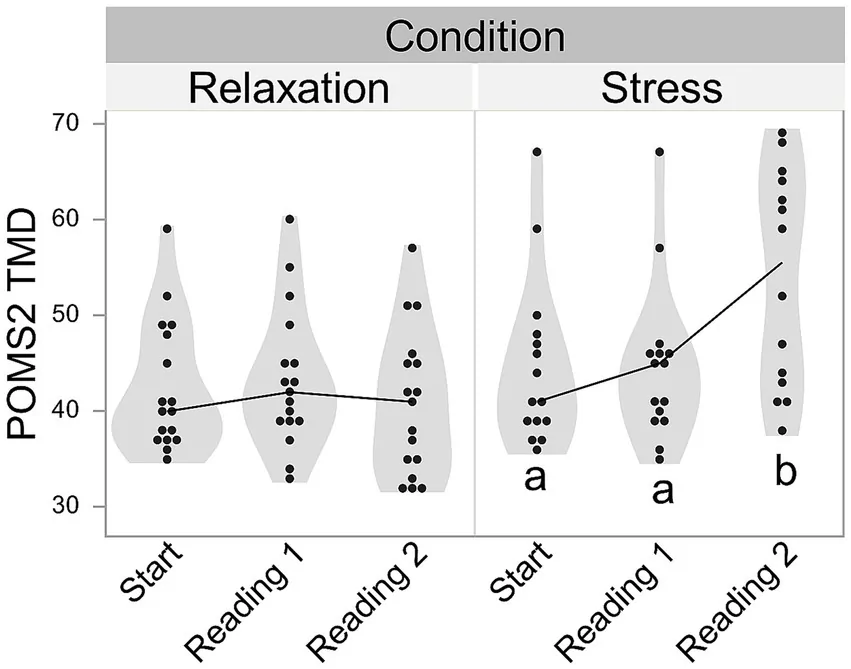
POMS2 TMD score. Violin plots show the distribution of scores. Within each panel, the groups that did not share a common letter differed significantly from one another.

Subjective mood states assessed using the POMS TMD scores also varied across the periods. The GLMM selected the full model as the best, with a significant effect of Period (*F* = 6.896, df1 = 2, df2 = 98, *p* = 0.002) and a significant interaction of Condition × Period (*F* = 20.352, df1 = 2, df2 = 98, *p* < 0.001). A stratified GLMM of the stress condition showed a significant effect of Period (*F* = 19.619, df1 = 2, df2 = 50, *p* < 0.001). Subsequent pairwise comparisons showed that the TMD scores for Reading 2 were higher than those for Reading 1 (*t* = 5.363, *p* < 0.001) and Rest 1 (*t* = 5.137, *p* < 0.001); however, no difference was observed between Reading 1 and Rest 1 (*t* = 0.254, *p* = 0.798). A stratified GLMM of the relaxation condition selected the null model as the best, indicating no differences across the periods (Figure 4 and Supplementary Table S7).

### 3.2 Dogs’ physiological and behavioral responses

**Figure 5:**
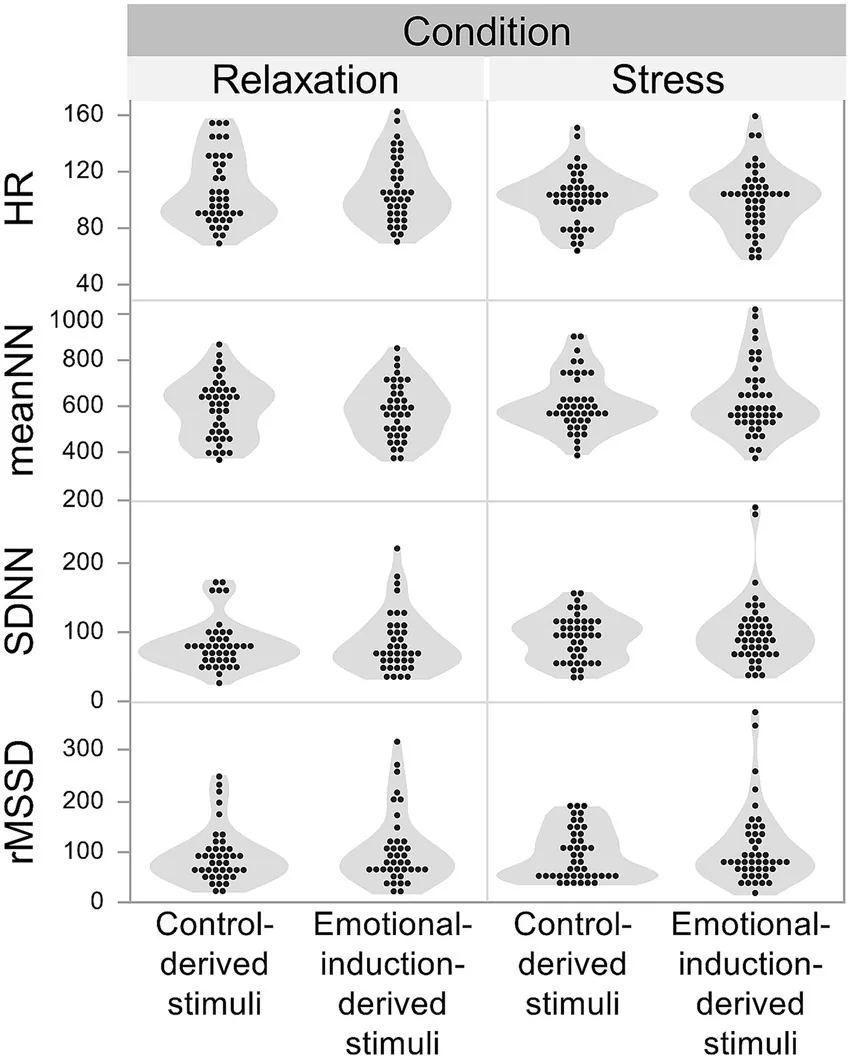
Dog HRV. Violin plots show the distributions of HRV indices.

In dogs, no effects of Condition or Stimulus-type were detected in HRV measures. Model selection based on the AIC consistently favored the null model over models that included fixed effects, indicating that dog HRV did not differ across experimental conditions or stimulus types. All HRV data for dogs are shown in Figure 5.

**Figure 6:**
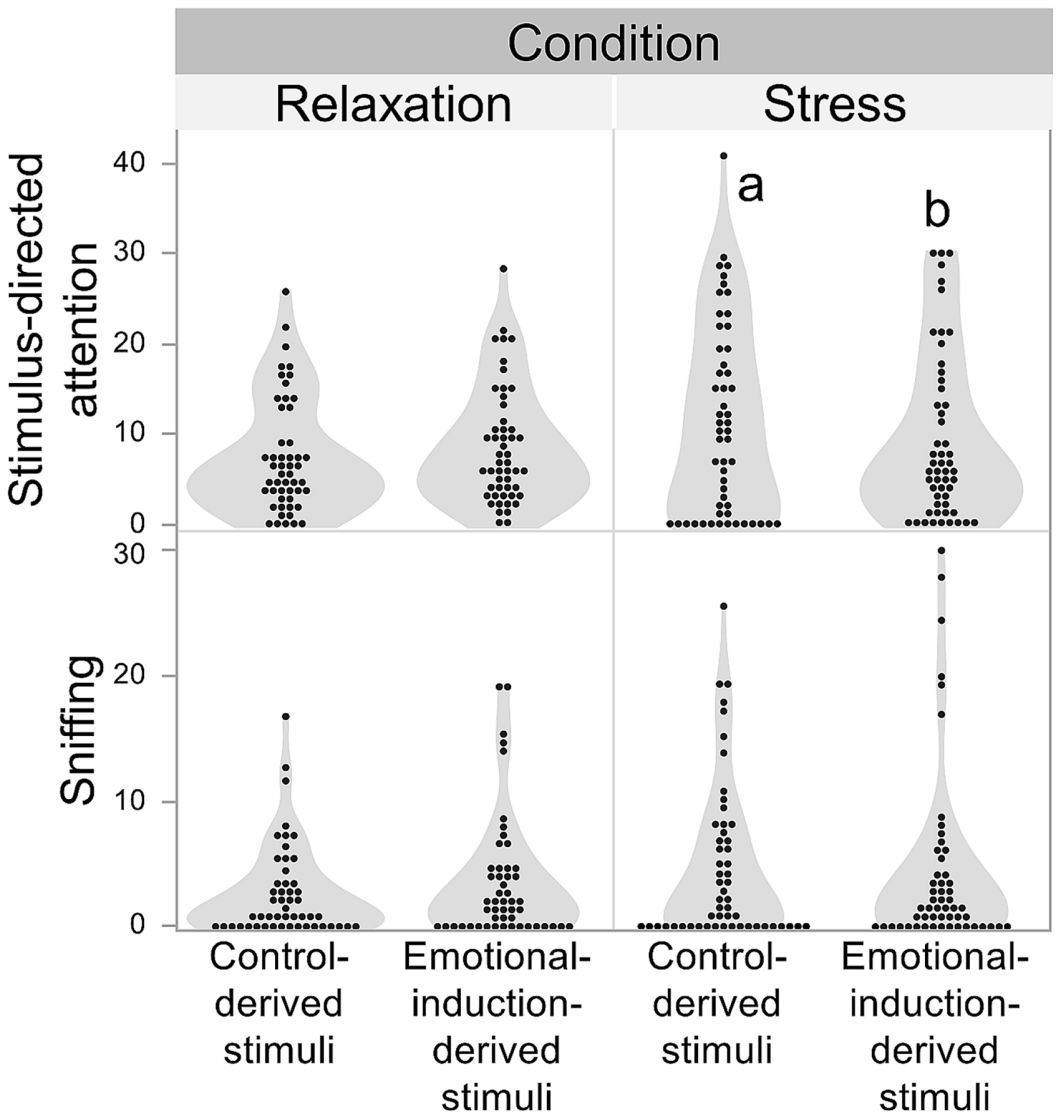
Dog’s behavior violin plots show the distributions of the durations of behaviors. Different letters indicate statistically significant differences.

In contrast, behavioral analyses were conducted using data from all 35 dogs, and several behavioral measures showed condition-dependent effects. For stimulus-directed attention, the GLMM selected the full model as the best model with a significant Condition × Stimulus-type interaction (*F* = 6.501, df1 = 1, df2 = 185, *p* = 0.012) but with no significant main effects. Follow-up GLMM conducted separately for each condition showed a significant effect of Stimulus type in the stress condition (*F* = 5.611, df1 = 1, df2 = 89, *p* = 0.020), but no significance in the relaxation condition. Pairwise comparisons showed that stimulus-directed attention was longer toward control-derived stimuli than toward stress-derived stimuli under the stress condition (*t* = 2.179, *p* = 0.032). A similar pattern was observed for the sniffing behavior. The GLMM analysis selected the full model as the best with a significant Condition × Stimulus-type interaction (*F* = 6.843, df1 = 1, df2 = 137, *p* = 0.010), but with no main effects. Stratified GLMMs separated for each condition revealed a significant effect of Stimulus-type in the stress condition (*F* = 4.165, df1 = 1, df2 = 69, *p* = 0.045), but no effects in the relaxation condition. Pairwise comparisons showed that dogs tended to sniff control-derived stimuli more than stress-derived stimuli under stress conditions (*t* = 1.860, *p* = 0.067). For standing behavior, the GLMM selected a model that included only the Condition with a significant effect (*F* = 4.930, df1 = 1, df2 = 200, *p* = 0.028). Subsequent pairwise comparisons revealed that standing was less in the stress condition than the relaxation condition (*t* = −2.176, *p* = 0.031). Other behaviors did not show significant results. The results are shown in Figure 6 and Supplementary Tables S8–S11.

For the remaining behavioral measures, analyses resulted in the null model being selected as the best model or in no significant main effects or interactions within the best model.

### 3.3 Third-party evaluation of owner-derived stimuli

A GLMM of third-party ratings of emotional valence selected the null model as the best, indicating that valence ratings did not differ across the experimental conditions. In contrast, the GLMM for arousal ratings selected a model that included only the Condition with a significant effect (*F* = 5.802, df1 = 1, df2 = 46, *p* = 0.020). Follow-up pairwise comparisons showed that ratings in the stress condition were lower than those in the relaxation condition (*t* = 2.444, *p* = 0.018) (Supplementary Table S12).

### 3.4 Correlation analysis between owner and dog HRV

**Figure 7:**
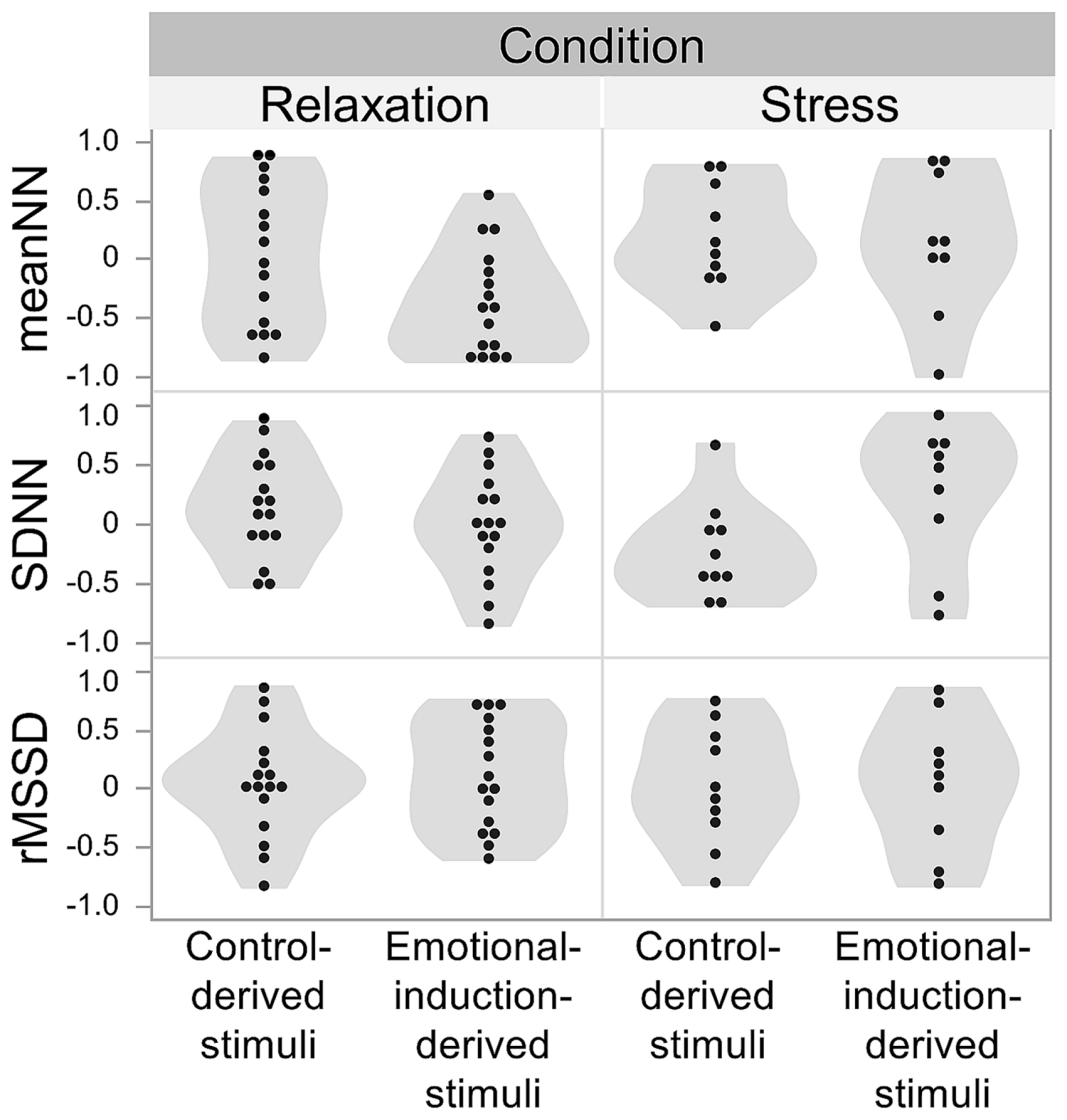
Comparison of correlation coefficients between owner and dog HRV indices.

The correlation coefficients between owner and dog HRV were further analyzed using LMMs. For meanNN, GLMM analysis showed that the full model was the best, with a marginal effect of Condition (*F* = 3.531, df1 = 1, df2 = 47, *p* = 0.066), but no significance of Stimulus-origin or Condition × Stimulus-origin interaction. Follow-up pairwise comparisons revealed that mean NN correlation coefficients tended to be lower in the stress condition than in the relaxation condition (*t* = −1.879, *p* = 0.066). For SDNN, GLMM analyses selected the full model as the best model with a significant Condition × Stimulus-origin interaction (*F* = 5.720, df1 = 1, df2 = 47, *p* = 0.021), but with no main effects. Stratified GLMMs conducted separately for each condition showed that the null model was the best model in the relaxation condition. However, the GLMM in the stress condition exhibited a marginal effect of Stimulus-origin (*F* = 4.340, df1 = 1, df2 = 17, *p* = 0.053). Subsequent pairwise comparisons showed that SDNN correlation coefficients tended to be lower for control-derived stimuli than for stress-derived stimuli (*t* = −2.083, *p* = 0.053). For the rMSSD, the null model was selected as the best model. All data are shown in Figure 7. The full results of the GLMM analyses of meanNN and SDNN are presented in Supplementary Tables S13, S14.

## 4 Discussion

This study investigated how dogs respond to experimentally induced, owner-derived emotional stimuli, focusing on behavioral responses, physiological responses (HRV), and owner–dog physiological synchrony. The following three main findings were obtained:

First, a dissociation was observed between owners’ physiological responses and subjective ratings of emotional states during stress induction.

Second, dogs exhibited context-dependent behavioral responses, showing greater attention toward stimuli derived from the control period than toward those derived from the emotional induction period in the stress condition.

Third, although dogs did not show differential physiological responses to owner-derived stimuli, synchrony in HRV between owners and dogs tended to increase under stressful conditions.

### 4.1 Dissociation between physiological and subjective responses in owners

Contrary to expectations, no significant differences in HRV were observed between the control and emotional induction periods under stressful conditions. However, both periods showed elevated heart rates and reduced meanNN compared to the rest, indicating increased physiological arousal relative to baseline. In contrast, the subjective mood (POMS TMD) increased significantly during the emotional induction period, confirming the successful induction of psychological stress.

This dissociation between physiological and subjective responses has been widely reported in stress research, where subjective experience does not necessarily align with physiological indices, such as cortisol or heart rate (32). One plausible explanation for the present findings is the influence of the reading task, which may have modulated autonomic activity through speech-related motor demands and respiratory patterns, thereby reducing detectable physiological differences between the control and emotional induction periods (33, 34).

Originally, the control period was intended to provide a relatively neutral comparison for the emotional induction period. However, the physiological findings indicated that the control-derived stimuli were not physiologically neutral, as originally intended. Rather, the control period may have reflected the physiological arousal associated with task engagement, despite the relatively low subjective stress reported by the owners.

In this study, physiological arousal during the control period was higher than that during the resting period and comparable to that during stress induction, whereas the subjective evaluation was less negative than that during the stress induction period. This dissociation between physiological responses and subjective evaluations observed during the control period has important implications for interpreting the behavioral responses of the dogs to the presented stimuli. Therefore, rather than representing a true neutral baseline, the control-derived stimuli should be interpreted as complex owner-derived multimodal stimuli reflecting different combinations of subjective emotional states, physiological arousal, and task-related influences.

### 4.2 Dogs’ behavioral responses to owner-derived stimuli

A key finding was that dogs in the stress condition displayed greater attentive behavior toward control-derived than stress-derived stimuli. Sniffing showed a similar tendency, although the effect was not statistically significant. Previous studies have suggested that dogs often reduce their attention or avoid negative emotional signals (22, 35, 36). Although our results could be interpreted as reflecting the avoidance of stress-derived stimuli, stress-related behaviors (such as licking, body shaking, and yawning) were rarely observed during stimulus presentation, and no clear condition-related changes in HRV among dogs were detected. These findings suggest that the stimuli did not elicit strong emotional arousal in the dogs, indicating that avoidance alone may not fully explain the observed behavioral pattern.

Another possible explanation is that the control-derived stimuli contained information that was more ambiguous or difficult for dogs to interpret than the stress-derived stimuli. Although owners exhibited elevated physiological arousal under both control and stress conditions, subjective emotional evaluations differed between them. Consequently, the control-derived stimuli may have contained less consistent information across available cues, requiring additional information gathering. Therefore, increased attentive behavior may have reflected an enhanced investigation of ambiguous social signals, rather than stronger emotional responses.

This interpretation is consistent with those of studies using cross-modal expectancy–violation paradigms, which have demonstrated increased attention toward incongruent stimuli in dogs (37, 38). Although such paradigms have not yet been directly applied to emotion perception in dogs (39), evidence from other species (40) has suggested that mismatches between emotional cues can promote exploratory and attentional behaviors. In this study, the combination of elevated physiological arousal and relatively low subjective stress in the control-derived stimuli may have produced a more ambiguous signal than that in the stress-derived stimuli, thereby eliciting greater information-gathering behavior.

Such information-gathering behavior may also be interpreted within the framework of active inference, in which additional information is acquired to reduce uncertainty. However, this experiment was not designed to distinguish active inferences from other possible explanations such as the investigation of ambiguous stimuli or partial avoidance of negative cues. Therefore, active inference should be regarded as a possible theoretical interpretation, rather than a demonstrated cognitive mechanism.

Methodological factors may also have contributed to the observed behavioral patterns. Although Albuquerque et al. (41) reported increased attention toward congruent emotional cues using simultaneous presentation and choice paradigms, in this study, we employed multimodal stimuli, including olfactory cues derived from subtle, everyday emotional expressions, rather than overt, experimentally exaggerated displays, thereby increasing stimulus complexity. As suggested by Correia-Caeiro et al. (39), such stimuli may substantially influence responses in dogs, leading to increased exploratory behavior when available information is less consistent. As visual, auditory, and olfactory cues were presented simultaneously, it is possible that subtle differences in one or more modalities contributed to the observed behavioral differences.

This interpretation is also consistent with the absence of behavioral differences in the relaxation conditions. Negativity bias, the preferential allocation of attention to negative stimuli (42), has also been reported in dogs responding to human emotional signals (41). In this study, under stress conditions, dogs may therefore have been more strongly motivated to gather information about the emotional states of their owners, increasing their sensitivity to subtle inconsistencies in the available cues. In contrast, the relaxation condition did not include negative or potentially threatening signals, which may have reduced the motivation for such a detailed investigation, despite the elevated heart rate associated with the reading task in the control-derived stimuli (Reading 1) to rest. Finally, third-party human evaluators did not detect differences in valence between the stimuli, and differences in arousal were only detectable at the condition level. Therefore, the behavioral differences observed in dogs suggest that they may have responded to subtle owner-derived multimodal cues that were not readily identified by unfamiliar human observers. However, our data did not allow us to determine which specific sensory cues or cognitive mechanisms underlay these behavioral responses.

Dogs in the stress condition also spent less time standing than those in the relaxation condition. Although this finding indicates that overall behavioral responses differed between emotional contexts, standing represents a relatively general postural measure, and its functional significance remains unclear in our experimental setting. Therefore, we considered stimulus-directed attention to be a more informative indicator of the responses of dogs to owner-derived stimuli.

### 4.3 Owner–dog physiological synchrony under stress

Correlation analyses were conducted between the HRV of the owners measured during the post-intervention reading periods (i.e., after the control and emotional induction periods) and that of the dogs recorded during the corresponding stimulus presentation periods. The results revealed that the correlation coefficients for the SDNN tended to be higher for stimuli derived from the emotional induction period under the stress condition. This pattern is consistent with previous findings (19) and suggests that physiological synchrony between owners and dogs may vary depending on the emotional context of owner-derived stimuli. However, as no clear effects of condition or stimulus type on dog HRV were detected, these findings do not provide direct evidence of emotional sharing or contagion.

Taken together with the behavioral findings, these results raise the possibility that dogs respond to owner-derived stimuli through multiple processes involving both behavioral and physiological components. One possible interpretation is that in stress-related contexts, dogs allocate greater behavioral resources to gathering information about their owners, while also displaying coordinated physiological responses. However, further studies are required to determine whether these physiological responses reflect emotional sharing, contagion, social bonding, or other underlying mechanisms.

### 4.4 Limitations and future directions

One important limitation concerns the nature of the control-derived stimuli. The control period was originally intended to provide a relatively neutral comparison with the emotional induction period. However, the HRV results suggest that the reading task itself elicited physiological arousal, indicating that the control-derived stimuli were not physiologically neutral as originally intended. Therefore, the control-derived stimuli should be regarded as complex owner-derived multimodal stimuli reflecting a combination of subjective emotional states, physiological arousal, and task-related influences. This complexity should be considered when interpreting the behavioral responses of the dogs. Future studies should employ valid stress-induction paradigms that minimize confounding factors, such as speech-related autonomic modulation.

Another limitation was the sample size. Although 35 owner–dog pairs participated in this study, the exclusion of HRV recordings due to missing or poor-quality data further reduced the sample size available for some physiological analyses. Consequently, the statistical power, particularly for detecting subtle physiological effects, may have been limited, and some true effects may have remained undetected. Future studies with larger sample sizes and more complete physiological datasets are needed to confirm the robustness of our findings.

In addition, it should be noted that visual and auditory stimuli were obtained during the fixed-sentence reading period that followed the intervention, rather than during emotional induction itself. In contrast, olfactory samples were collected during both the intervention and subsequent reading periods. Therefore, the extent to which the presented stimuli accurately reflect the same emotional state remains unclear. Furthermore, because the visual, auditory, and olfactory cues were presented simultaneously, the relative contribution of each sensory modality could not be determined. Accordingly, the observed behavioral responses should be interpreted as responses to composite owner-derived stimuli, rather than to any individual sensory cue.

Moreover, although all predefined behavioral variables were analyzed, significant effects were observed only for a subset of behaviors. Therefore, these findings should be interpreted with caution until they are replicated in larger, independent samples.

Despite these limitations, the relatively subtle and complex nature of owner-derived stimuli provides an opportunity to investigate the responses of dogs to emotionally relevant multimodal cues encountered in everyday interactions, rather than to overt emotional expressions. The combined pattern of subjective differences, behavioral responses of dogs, and the tendency toward physiological synchrony is consistent with the possibility that dogs respond to subtle owner-derived cues. However, this study did not identify specific sensory cues or cognitive mechanisms underlying these responses. Nevertheless, our findings highlight the importance of using multilevel approaches to investigate interspecific emotional communication.

## 5 Conclusion

Overall, these results suggest that dogs exhibit differential behavioral responses to owner-derived multimodal stimuli associated with different combinations of subjective emotional states, physiological arousal, and task-related influences. Although no clear condition-related effects were detected in the HRV of dogs, differences in stimulus-directed attention and the tendency toward stronger owner–dog physiological synchrony under stress suggested that dogs may respond to subtle variations in owner-derived cues. However, as we employed complex multimodal stimuli and did not isolate the contributions of individual sensory modalities or cognitive mechanisms, our findings should be interpreted with caution. Further studies using larger sample sizes and experimentally controlled stimuli are necessary to clarify the mechanisms underlying the responses of dogs to subtle owner-derived emotional cues.

## Data Availability

The raw data supporting the conclusions of this article will be made available by the authors, without undue reservation.
