# Supplementary material for: Dogs display context-dependent behavioral responses to subtle owner-derived multimodal stimuli
Source: Front Vet Sci. 2026 Aug 20;13:1916007. doi: 10.3389/fvets.2026.1916007 (PMC13540256; doi:10.3389/fvets.2026.1916007)
Supplement: Supplementary file 1 [file Data_Sheet_1.pdf]

Table S1. Information of dogs and owners

Condition	Breed	Dog sex	Neuter status	Dog age (months)	Owner sex	Owner age (years)
Stress	Pug	female	neutered	48	female	51
	Labrador Retriever	female	neutered	38	female	36
	Poodle (toy)	male	neutered	15	female	49
	Poodle (toy)	male	neutered	28	female	49
	Miniature Bull Terrier	female	neutered	100	female	48
	Shiba-inu	male	neutered	35	female	56
	Mongrel	female	neutered	122	female	55
	Bichon Frise	male	intact	55	female	55
	Italian Greyhound	male	neutered	86	female	41
	West Highland White Terrier	male	neutered	28	female	62
	Mixed	male	neutered	122	female	58
	Pomeranian	female	neutered	55	female	53
	Shiba-inu	male	neutered	59	male	60
	Boston Terrier	male	neutered	27	female	47
	Shiba-inu	male	neutered	110	female	50
	Labrador Retriever	male	nuetered	38	female	50
	Afghan_Hound	male	nuetered	24	female	62
	Mixed	male	nuetered	30	female	36
Relaxation	Mixed	male	neutered	21	female	55
	Shiba-inu	male	intact	71	female	36
	Mongrel	male	neutered	73	female	35
	Poodle (standard)	female	neutered	54	female	63
	Poodle (toy)	male	neutered	117	female	53
	Poodle (toy)	female	neutered	12	female	38
	Shetland Sheepdog	female	neutered	72	male	40
	Shiba-inu	male	neutered	15	female	49
	Pomeranian	female	intact	16	female	64
	Pomeranian	female	intact	15	female	64
	Shetland Sheepdog	female	intact	13	female	34
	Italian Greyhound	female	neutered	47	female	41
	Italian Greyhound	female	neutered	22	female	41
	Pomeranian	male	neutered	27	male	65
	Mixed	female	neutered	42	female	53
	Shiba-inu	female	neutered	79	female	54
	Akita-inu	male	neutered	72	female	54

Table S2. Definition of dog's behavior

Behavioral category	Behavior	Definition
State behaviors	Moving	Walking or locomotion; defined by at least two steps within one second. The duration is measured from the moment the first paw leaves the ground until the last moving paw returns to a stationary position.
	Standing	Stationary quadrupedal posture with all four paws in contact with the ground and no visible trunk movement.
	Sitting	Sitting posture; the duration begins when the dog ' s hindquarters make contact with the floor and ends when they leave the floor.
	Down	Recumbent (lying) posture; begins when the dog ' s abdomen makes contact with the floor and ends when it is lifted.
	Up	Bipedal posture; the dog is supported only by its hind legs with both forelegs lifted off the ground.
	Tail-high	The majority of the tail, including the tip, is held at or above the dorsal line (the line of the back).
	Tail-low	The majority of the tail is held below the dorsal line.
	Tail-wagging	Rhythmic lateral movement of the tail. Movements resulting passively from body shakes are excluded.
	Stimulus-directed attention	Active engagement with the presented stimulus, operationally defined as directing visual attention toward the monitor and/or actively investigating the stimulus device. Sniffing represents a specific subtype of stimulus-directed attention and was additionally coded separately to quantify active olfactory exploratory behavior.
	Sniffing	A specific sub-category of stimulus-directed attention, defined as active olfactory exploration with the mouth closed and the nose directed toward stimulus-related targets (e.g., the floor, odor bottle, or surrounding air). This excludes incidental nasal movements during open-mouthed breathing and was coded separately to quantify active olfactory investigation.
	Whining	High-pitched, often nasal, vocalization.
Event behaviors	Body shaking	Rapid, vigorous oscillation of the trunk and head. One event is recorded from the onset of movement until the body returns to a stationary state.
	Yawning	Opening and closing of the mouth in a single deep inhalation/exhalation cycle.
	Licking	Visible contact of the tongue with the nose or lips. Simple tongue protrusions without contact are excluded.

Table S3. Summary of model selections

Dependent variable	Smaple size	Model selection
**HRVs in Owner Phase**		
HR	103	Table S4
meanNN	103	Table S5
SDNN	103	Table S6
rMSSD	103	Null model was selected. (AIC_null = 17.09 , AIC_full = 31.54)
**POMS in Owner Phase**		
POMS	104	Table S7
**Behavior in Dog phase**		
Stimulus-directed attention	189	Table S8
Sniffing	141	Table S9
Standing	202	Table S10
Whining	93	Table S11
Sitting	45	Table S11
Down	16	Table S11
Up	13	Table S11
Tail-high	210	Null model was selected. (AIC_null = 5898.49 , AIC_full = 5947.70)
Tail-low	210	Null model was selected. (AIC_null = 4776.56, AIC_full = 4954.69)
Tail-wagging	210	Null model was selected. (AIC_null = 7361.44, AIC_full = 7574.34)
Moving	134	Null model was selected. (AIC_null = 298.92, AIC_full = 303.12)
Body shaking	Not observed	-
Yawning	Not observed	-
Licking	Not observed	-
**HRVs in Dog Phase**		
HR	170	Null model was selected. (AIC_null= -214.56, AIC_full = -200.19)
meanNN	170	Null model was selected. (AIC_null = -231.78, AIC_full = -217.13)
SDNN	170	Null model was selected. (AIC_null = 119.98, AIC_full = 128.47)
rMSSD	170	Null model was selected. (AIC_null = 209.23, AIC_full = 214.81)
**SAM of the Third Party**		
Arousal	48	Table S12
Emotional Valence	48	Null model was selected. (AIC_null= -14.538, AIC_full = -7.792)
**Correlation coefficients of HRVs between dog and owner**		
meanNN	170	Table S13
SDNN	170	Table S14
rMSSD	170	Null model was selected. (AIC_null = 209.23, AIC_full = 214.81)

Table S4. Fixed effects of the best-fitted model to HR in Owner Phase

Best model (AIC_best (full) = -253.50, AIC_null = -251.63)

n = 103 Marginal R^2 = 0.119, Conditional R^2 = 0.707

Effect	*F*	*df1*	*df2*	*p*
Condition	0.158	1	95	0.692
Period	9.81	3	95	<0.001
Condition x Period	9.437	3	95	<0.001

Stratified model: Stress condition (AIC_best (full) = -96.31, AIC_null = -94.69)

n = 41 Marginal R^2 = 0.144, Conditional R^2 = 0.676

Effect	*F*	*df1*	*df2*	*p*
Period	7.537	3	37	<0.001

Pairwise comparisons (Stress condition)

Comparison	*Estimate*	*SE*	*t*	*df*	*p_adj*
Control - Emotion	0.307	1.316	0.233	37	0.817
Control - Rest 2	3.989	1.349	2.957	37	0.005
Control - Rest 1	4.793	1.286	3.725	37	0.001
Emotion - Rest 2	3.682	1.328	2.772	37	0.009
Emotion - Rest 1	4.486	1.237	3.627	37	0.001
Rest 2 - Rest 1	0.804	1.291	0.623	37	0.537

Stratified model: Relaxation condition (AIC_best (full) = -155.36, AIC_null = -147.62)

n = 62 Marginal R^2 = 0.109, Conditional R^2 = 0.724

Effect	*F*	*df1*	*df2*	*p*
Period	12.265	3	58	<0.001

Pairwise comparisons (Relaxation condition)

Comparison	*Estimate*	*SE*	*t*	*df*	*p_adj*
Control - Emotion	5.679	1.019	5.575	58	< 0.001
Control - Rest 2	4.104	0.983	4.174	58	< 0.001
Control - Rest 1	2.023	0.991	2.041	58	0.046
Emotion - Rest 2	-1.575	0.981	-1.606	58	0.114
Emotion - Rest 1	-3.657	0.998	-3.663	58	0.001
Rest 2 - Rest 1	-2.082	0.965	-2.158	58	0.035

Table S5. Fixed effects of the best-fitted model to meanNN in Owner Phase

Best model (AIC_best (full) = -254.45, AIC_null = -252.77)

n = 103 Marginal R^2 = 0.119, Conditional R^2 = 0.707

Effect	*F*	*df1*	*df2*	*p*
Condition	0.159	1	95	0.691
Period	9.917	3	95	<0.001
Condition x Period	9.499	3	95	<0.001

Stratified model: Stress condition (AIC_best (full) = -96.49, AIC_null = -95.19)

n = 41 Marginal R^2 = 0.144, Conditional R^2 = 0.677

Effect	*F*	*df1*	*df2*	*p*
Period	7.674	3	37	<0.001

Pairwise comparisons (Stress condition)

Comparison	*Estimate*	*SE*	*t*	*df*	*p_adj*
Control - Emotion	-4.096	12.704	-0.322	37	0.749
Control - Rest 2	40.978	13.715	2.988	37	0.005
Control - Rest 1	-50.274	13.126	-3.83	37	< 0.001
Emotion - Rest 2	-36.882	13.601	-2.712	37	0.01
Emotion - Rest 1	-46.178	12.727	-3.628	37	0.001
Rest 2 - Rest 1	-9.296	13.913	-0.668	37	0.508

Stratified model: Relaxation condition (AIC_best (full) = -155.69, AIC_null = -148.37)

n = 62 Marginal R^2 = 0.109, Conditional R^2 = 0.724

Effect	*F*	*df1*	*df2*	*p*
Period	12.274	3	58	<0.001

Pairwise comparisons (Relaxation condition)

Comparison	*Estimate*	*SE*	*t*	*df*	*p_adj*
Control - Emotion	-60.111	10.856	-5.537	58	< 0.001
Control - Rest 2	-42.359	10.15	-4.142	58	< 0.001
Control - Rest 1	-19.584	9.996	-1.959	58	0.055
Emotion - Rest 2	17.872	10.989	1.626	58	0.109
Emotion - Rest 1	40.527	10.9	3.718	58	< 0.001
Rest 2 - Rest 1	22.655	10.272	2.206	58	0.031

Table S6. Fixed effects of the best-fitted model to SDNN in Owner Phase

Best model (AIC_best = -22.61, AIC_full = -15.67, AIC_null = -16.77)

n = 103   Marginal R^2 = 0.072, Conditional R^2 = 0.597

Effect	*F*	*df1*	*df2*	*p*
Period	7.861	3	99	<0.001

Pairwise comparisons

Comparison	*Estimate*	*SE*	*t*	*df*	*p_adj*
Control - Emotion	-4.261	1.427	-2.986	99	0.004
Control - Rest 2	-6.594	1.494	-4.414	99	< 0.001
Control - Rest 1	-1.953	1.331	-1.468	99	0.145
Emotion - Rest 2	-2.332	1.565	-1.49	99	0.139
Emotion - Rest 1	2.309	1.433	1.611	99	0.11
Rest 2 - Rest 1	4.641	1.503	3.087	99	0.003

Table S7. Fixed effects of the best-fitted model to POMS in Owner Phase

Best model (AIC_best (full) = -92.28, AIC_null = -66.24)

n = 104 Marginal R^2 = 0.221, Conditional R^2 = 0.658

Effect	*F*	*df1*	*df2*	*p*
Condition	2.629	1	98	0.108
Period	6.896	2	98	<0.001
Condition x Period	20.352	2	98	<0.001

Stratified model: Stress condition (AIC_best (full) = -35.53, AIC_null = -11.28)

n = 53 Marginal R^2 = 0.068, Conditional R^2 = 0.505

Effect	*F*	*df1*	*df2*	*p*
Period	7.674	3	37	<0.001

Pairwise comparisons (Stress condition)

Comparison	*Estimate*	*SE*	*t*	*df*	*p_adj*
Control - Emotion	-9.746	1.817	-5.363	50	< 0.001
Control - Rest 1	-0.399	1.549	-0.257	50	0.798
Emotion - Rest 1	9.348	1.819	5.137	50	< 0.001

Stratified model: Relaxation condition (AIC_best (null) = -65.30, AIC_full = -59.02)

n = 51

(Null model was selected.)

Table S8. Fixed effects of the best-fitted model to Stimulus-directed attention in Dog phase

Best model (AIC_best (full) = 430.28, AIC_null = 438.20)

n = 189 Marginal R^2 = 0.062, Conditional R^2 = 0.288

Effect	*F*	*df1*	*df2*	*p*
Condition	2.474	1	185	0.117
Stimulus-type	0.988	1	185	0.322
Condition x Stimulus-type	6.501	1	185	0.012

Stratified model: Stress condition (AIC_best (full) = 217.72, AIC_null = 223.44)

n = 91 Marginal R^2 = 0.043, Conditional R^2 = 0.308

Effect	*F*	*df1*	*df2*	*p*
Stimulus-type	5.611	1	89	0.020

Pairwise comparisons (Stress condition)

Comparison	*Estimate*	*SE*	*t*	*df*	*p_adj*
Control-derived stimuli – Emotional-induction-derived stimuli	3.353	1.539	2.179	89	0.032

Stratified model: Relaxation condition (AIC_best (null) = 215.57, AIC_full = 216.71)

n = 98

(Null model was selected.)

Table S9. Fixed effects of the best-fitted model to sniffing behavior in Dog phase

Best model (AIC_best (full) = 389.36, AIC_null = 395.22)

n = 141 Marginal R^2 = 0.004, Conditional R^2 = 0.348

Effect	*F*	*df1*	*df2*	*p*
Condition	1.051	1	137	0.307
Stimulus-type	0.002	1	137	0.964
Condition x Stimulus-type	0.010	1	137	0.010

Stratified model: Stress condition (AIC_best (full) = 198.02, AIC_null = 201.81)

n = 71 Marginal R^2 = 0.036, Conditional R^2 = 0.422

Effect	*F*	*df1*	*df2*	*p*
Stimulus-type	5.611	1	89	0.020

Pairwise comparisons (Stress condition)

Comparison	*Estimate*	*SE*	*t*	*df*	*p_adj*
Control-derived stimuli – Emotional-induction-derived stimuli	1.848	0.993	1.860	69	0.067

Stratified model: Relaxation condition (AIC_best (full) = 193.30, AIC_full = 197.30)

n = 70 Marginal R^2 = 0.037, Conditional R^2 = 0.213

Effect	*F*	*df1*	*df2*	*p*
Stimulus-type	2.835	1	68	0.097

Table S10. Fixed effects of the best-fitted model to standing behavior in Dog phase

Best model (AIC_best = 181.79, AIC_full = 188.90, AIC_null = 183.72)

n = 202 Marginal R^2 = 0.137, Conditional R^2 = 0.415

Effect	*F*	*df1*	*df2*	*p*
Condition	4.930	1	200	0.028

Pairwise comparisons

Comparison	*Estimate*	*SE*	*t*	*df*	*p_adj*
Relaxation – Stress	-6.173	2.837	-2.176	200	0.031

Table S11. Fixed effects of the best-fitted model to other behaviors in Dog phase

<Whining>

Best model (AIC_best = 255.47, AIC_full = 256.19, AIC_null = 256.17)

n = 93 Marginal R^2 = 0.009, Conditional R^2 = 0.535

Effect	*F*	*df1*	*df2*	*p*
Condition	0.165	1	91	0.685

<Sitting>

Best model (AIC_best = 116.77, AIC_full = 117.91, AIC_null = 117.72)

n = 45 Marginal R^2 = 0.001, Conditional R^2 = 0.597

Effect	*F*	*df1*	*df2*	*p*
Condition	0.105	1	42	0.749

<Up>

Best model (AIC_best(full) = 31.40, AIC_null = 36.57)

n = 13 Marginal R^2 = 0.208, Conditional R^2 = 0.745

Effect	*F*	*df1*	*df2*	*p*
Condition	4.350	1	7	0.075
Stimulus-type	0.001	1	7	0.978
Condition x Stimulus-t	0.684	1	7	0.435

<Down>

Best model (AIC_best(full) = 35.33, AIC_null = 36.57)

n = 16 Marginal R^2 = 0.144, Conditional R^2 = 0.856

Effect	*F*	*df1*	*df2*	*p*
Condition	0.280	1	14	0.605

Table S12. Fixed effects of the best-fitted model to arousal rating of SAM of the third party

Best model (AIC_best = 53.409, AIC_full = 56.257, AIC_null = 56.555)

n = 48 Marginal R^2 = 0.251, Conditional R^2 = 0.361

Effect	*F*	*df1*	*df2*	*p*
Condition	5.802	1	46	0.02

Pairwise comparisons

Comparison	*Estimate*	*SE*	*t*	*df*	*p_adj*
Relaxation - Stress	1.729	0.707	2.444	46	0.018

Table S13. Fixed effects of the best-fitted model to correlation coefficient of meanNN between dog and owner

Best model (AIC_best (full) = 84.18, AIC_null = 89.44)

n = 51 Marginal R^2 = 0.061, Conditional R^2 = 0.132

Effect	*F*	*df1*	*df2*	*p*
Condition	3.531	1	47	0.066
Stimulus-origin	2.999	1	47	0.090
Condition x Stimulus-origin	2.420	1	47	0.126

Pairwise comparisons (Condition)

Comparison	*Estimate*	*SE*	*t*	*df*	*p_adj*
Relaxation – Stress	-0.336	0.179	-1.879	47	0.066

Table S14. Fixed effects of the best-fitted model to correlation coefficient of SDNN between dog and owner

Best model (AIC_best (full) = 74.24, AIC_null = 75.13)

n = 51 Marginal R^2 = 0.027, Conditional R^2 = 0.073

Effect	*F*	*df1*	*df2*	*p*
Condition	0.146	1	47	0.704
Stimulus-origin	1.414	1	47	0.240
Condition x Stimulus-origin	5.720	1	47	0.021

Stratified model: Stress condition (AIC_best (full) = 33.78, AIC_null = 36.67)

n = 19 Marginal R^2 = 0.119, Conditional R^2 = 0.194

Effect	*F*	*df1*	*df2*	*p*
Stimulus-origin	4.340	1	17	0.053

Pairwise comparisons (Stress condition)

Comparison	*Estimate*	*SE*	*t*	*df*	*p_adj*
Control-derived stimuli – Emotional-induction-derived stimuli	-0.475	0.228	-2.083	17	0.053

Stratified model: Relaxation condition (AIC_best (null) = 43.99, AIC_full = 44.82)

n = 32

(Null model was selected.)
